# The influence of gravity and light on locomotion and orientation of *Heterocypris incongruens* and *Notodromas monacha* (Crustacea, Ostracoda)

**DOI:** 10.1038/s41526-017-0037-5

**Published:** 2018-01-18

**Authors:** Jessica Fischer, Christian Laforsch

**Affiliations:** 1 Animal Ecology I, University of Bayreuth, Universitaetsstrasse 30, 95447 Bayreuth, Germany; 2 Bayreuth Center of Ecology and Environmental Research (BayCEER), University of Bayreuth, Universitaetsstrasse 30, 95447 Bayreuth, Germany

## Abstract

For future manned long-d uration space missions, the supply of essentials, such as food, water, and oxygen with the least possible material resupply from Earth is vital. This need could be satisfied utilizing aquatic bioregenerative life support systems (BLSS), as they facilitate recycling and autochthonous production. However, few organisms can cope with the instable environmental conditions and organic pollution potentially prevailing in such BLSS. Ostracoda, however, occur in eu- and even hypertrophic waters, tolerate organic and chemical waste, varying temperatures, salinity, and pH ranges. Thus, according to their natural role, they can link oxygen liberating, autotrophic algae, and higher trophic levels (e.g., fish) probably also in such harsh BLSS. Yet, little is known about how microgravity (µ*g*) affects Ostracoda. In this regard, we investigated locomotion and orientation, as they are involved in locating mating partners and suitable microhabitats, foraging, and escaping predators. Our study shows that Ostracoda exhibit altered activity patterns and locomotion behavior (looping) in µ*g*. The alterations are differentially marked between the studied species (i.e., 2% looping in *Notodromas monacha*, ~50% in *Heterocypris incongruens*) and also the thresholds of gravity perception are distinct, although the reasons for these differences remain speculative. Furthermore, neither species acclimates to µ*g* nor orientates by light in µ*g*. However, Ostracoda are still promising candidates for BLSS due to the low looping rate of *N. monacha* and our findings that the so far analyzed vital functions and life-history parameters of *H. incongruens* remained similar as under normal gravity conditions despite of its high looping rate.

## Looping shrimp could still be part of space food chain

Despite disorientation in microgravity, seed shrimp could form part of the food chain in artificial ecosystems designed for space missions. Jessica Fischer and Christian Laforsch of Germany’s University of Bayreuth investigated how simulated microgravity changed the movement of two species of seed shrimp (Ostracoda). Ostracoda can cope in unstable conditions and are being considered as food for fish in aquatic bioregenerative life support systems (BLSS) in manned space missions. Fish fed on Ostracoda that eat algae in these systems could form an important source of proteins for humans. *Notodromas monacha* and *Heterocypris incongruens* moved around in loops when exposed to simulated microgravity, but *N. monacha* did not loop as much. Looping movements suggest that gravity is an important orientational cue. Previous studies showed Ostracoda survived and reproduced successfully for months in space despite their looping behavior. Further studies are needed on *N. monacha*, since its low looping behavior could mean lower energy consumption making it better qualified for BLSS.

## Introduction

For future manned long-duration missions as for example to the planet Mars, the sustained supply of essentials, such as food, water, and oxygen is vital. These needs could be satisfied utilizing bioregenerative life support systems (BLSS). Based on real ecosystems, they facilitate recycling and autochthonous production and are thus independent of the commonly practiced (i.e., on the International Space Station), but uneconomic material resupply.^1^

A large part of the so far designed and tested BLSS is based on photoautotrophic unicellular algae, as they are used in photobioreactors to revitalize the atmosphere by recycling carbon dioxide while producing oxygen^2^ In this process also algae biomass is produced, which could be exploited as food source.^3^ However, not all unicellular algae are suitable for human consumption and for a balanced diet and mind of astronauts, it is furthermore desirable to convert at least a portion of the edible algae biomass into animal protein.^4^

In aquatic ecosystems on Earth, microcrustaceans are a key link between algal primary production and higher trophic levels.^5^ Thus, they are perfect candidates to occupy this role in BLSS, where they could serve as food for fish,^6^ which in turn could provide the animal protein for astronauts.^7^ However, instable environmental conditions (e.g., oxygen concentration, pH value,^8,9^ and organic pollution^10,11^) render BLSS harsh habitats, precluding most microcrustacean taxa from a deployment in these systems.

Many Ostracoda are extremely well suited to face such challenges. They inhabit eu- and even hypertrophic waters^12,13^ and are able to tolerate organic and chemical waste (e.g., water-borne ammonia and phosphate,^14^) high temperatures, salinity, and pH ranges.^15^ Moreover, they can deploy conservation strategies, such as a temporary closure of their valves to overcome momentary prevailing detrimental conditions or the production of dormant eggs to cope with long-lasting unfavorable environmental conditions.^16,17^ Since these eggs have the ability to hatch in microgravity (µ*g*),^18^ a BLSS could be restarted at any point, for example after collapse.^19^ Another reason for the utilization of ostracods in BLSS is that many species are omnivorous.^12^ In case of a disruption of the algae supply, they can still subsist and furthermore potentially recycle remnant organic material.^20^ Nevertheless, still little is known about how µ*g* affects Ostracoda. Species preselected as result of their ecological preconditions thus have to be studied thoroughly under this condition.

One of the most important issues in this regard is locomotion. Due to its involvement in locating mating partners and suitable microhabitats, foraging, and escaping predators, it is essential for survival. Closely associated to a directed locomotion behavior is spatial orientation, as it enables organisms to control their position within their three-dimensional habitat by using the gravitational vector as reliable reference.^21^ However, only a single experiment, carried out on MIR space station, addressed locomotion and orientation of Ostracoda in µ*g* subordinated within the investigation of a closed ecological system.^22,23^ During a four-months long sojourn in space, the animals performed looping behavior,^22,23^ an abnormal (kinetotic) locomotion behavior. This behavior has already been observed in various aquatic organisms in µ*g* (ranging from Protozoa^24^ to invertebrates^25,26^ to vertebrates^27^) and is regarded to be a disorientation response. However, the observations were entirely made under white-light (WL) illumination. A distinction between the influence of light and gravity on orientation was therefore impossible. Besides looping behavior, all other types of locomotion and the impact of µ*g* on these behaviors (e.g., drifting instead of descending along the gravitational vector when the animals passively stay within water column) were neglected in this study. Moreover, the ostracod species deployed on the MIR space station remained unidentified, neglecting a species specificity in the reaction to µ*g* within this extremely diverse taxon. Although the MIR experiment indicates that Ostracoda may be well suited for the application in BLSS, as they survived and reproduced the whole space mission, there is still a considerable lack of knowledge on the behavior in µ*g* and the varieties among different species.

For the present analysis of locomotion behavior and orientation, we thus deployed two ostracod species, *Heterocypris incongruens* and *Notodromas monacha*, in order to cover possible variation in the susceptibility to and the performance in µ*g* within the taxon. Even though co-occurring in many waterbodies, they possess morphological, physiological, and behavioral differences due to a heterogeneous habitat use. *H. incongruens* lives in the benthic and pelagic zone,^28,29^ *N. monacha* is partially neustonic and turns its body upside down to cling to the water surface membrane or crawls on plants.^30^ Utilizing parabolic flights (PFs), a sounding rocket flight (TEXUS—Technologische Experimente unter Schwerelosigkeit) and clinorotation (clino), we investigated possible behavioral changes of these species in µ*g*, which could diminish their performance in BLSS. Furthermore, we analyzed, if such changes can be compensated or reduced. Therefore, we assessed the influence of illumination, which could provide a cue for spatial orientation in µ*g*, as other species use the direction of incident light for orientation^31,32^ and a possible acclimation to µ*g* over time. Moreover, we approached the threshold of gravity perception of both species under partial-gravity conditions.

## Results

### Comparison of locomotion behavior between normal and microgravity

**Fig. 1:**
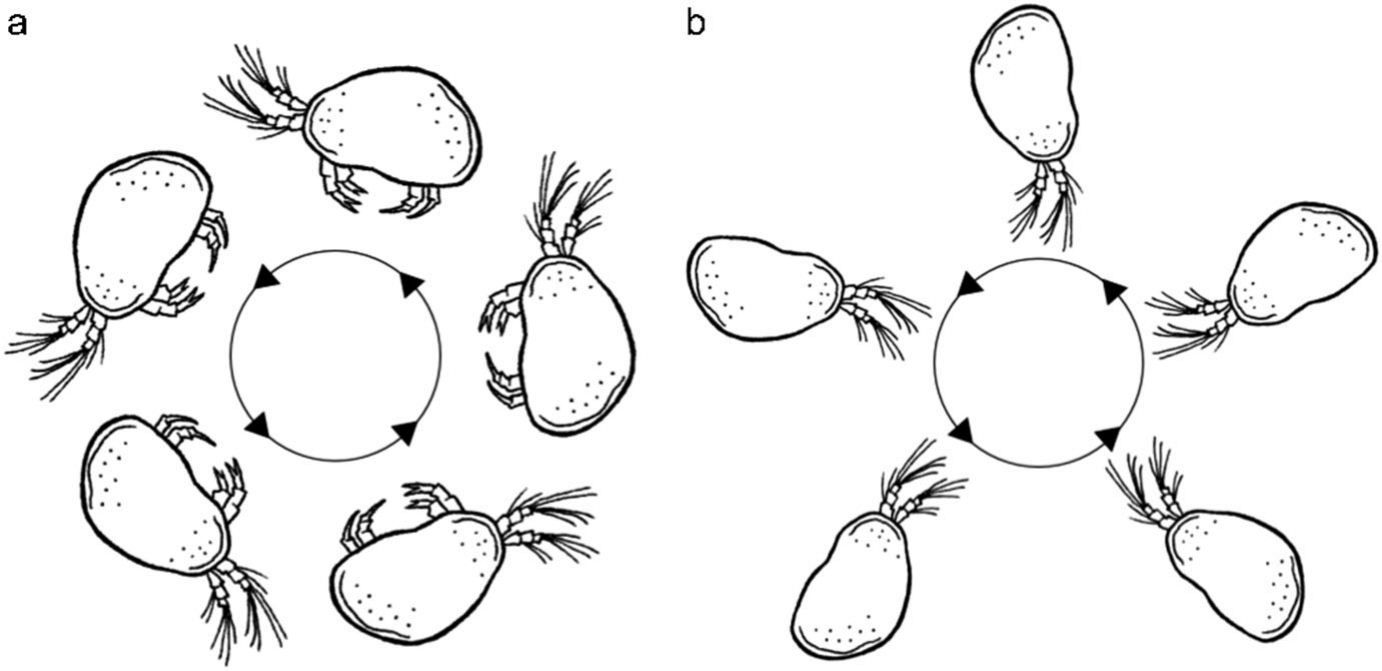
**a** Scheme of the µ*g*-specific looping behavior displayed by *H. incongruens* as well as *N. monacha*, during which the ventral side of the animals is pointing toward the center of the actively performed circles; **b** passive rotation of *H. incongruens* caused by clinorotation, when the ostracods show drifting behavior

Exclusively in µ*g* (real µ*g* during PFs [*H. incongruens* and *N. monacha*] and TEXUS [*H. incongruens*], simulated µ*g* during clino [*H. incongruens*]) both species looped (Fig. 1a) and drifted through the water column.

**Fig. 2:**
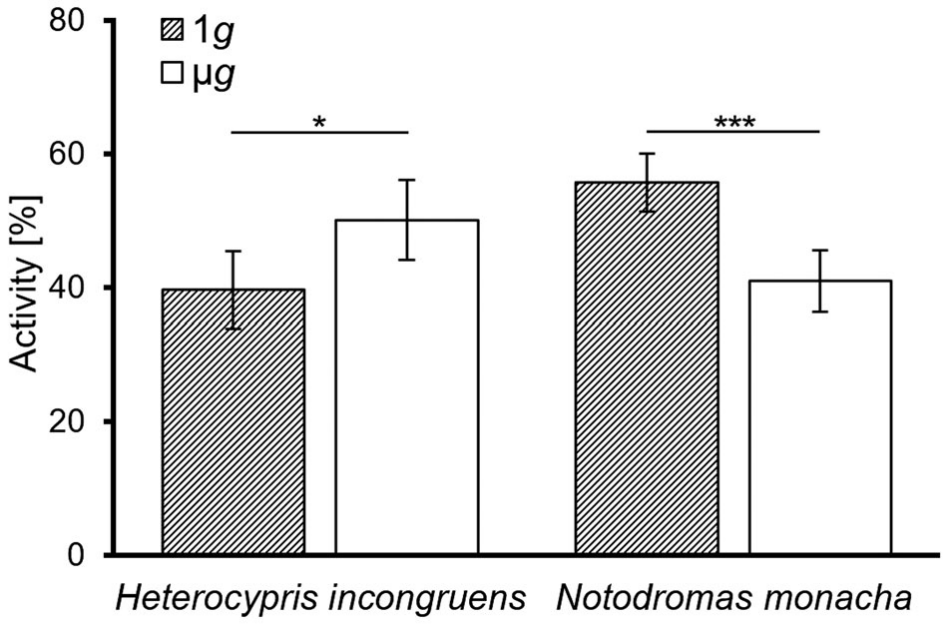
Relative activity (in %) of *H. incongruens* and *N. monacha* in 1*g* and µ*g* during parabolic flights. Mean value ± SEM, *n* = 18, levels of significance: *p ≤ 0.05; ***p ≤ 0.001

However, unlike in real µ*g* (PFs: 3.01 ± 0.95% of the time under infrared (IR) and 5.20 ± 1.50% under WL illumination; TEXUS: 7.78 ± 3.19%), *H. incongruens* drifted at a high proportion under clino (34.4 ± 9.4% of the time under IR and 22.92 ± 4.89% under WL illumination). Due to the rotation of the clinostat and the thus emerging forces, the animals were thereby forced on circular paths and passively aligned, so that the first antennae always pointed to the center of the circle around which they revolved (Fig. 1b). Clino thus not only altered the perceivable gravitational force, but also induced further forces affecting orientation and locomotion. During the PFs, however, no other force was altered between normal gravity (1*g*) and µ*g*, except the gravitational force. Thus, only differences in looping and drifting behavior and in activity (Fig. 2) observed during these flights were analyzed statistically.

*H. incongruens* as well as *N. monacha* looped in µ*g* significantly longer than in 1*g* (*H. incongruens*: *Z* = 3.72, *p* < 0.001; *N. monacha*: *Z* = 2.37, *p* = 0.018). *H. incongruens* also drifted significantly more in µ*g* (*Z* = 2.93, *p* = 0.003), in *N. monacha* the difference is not significant (*Z* = 1.34, *p* = 0.180). All other behaviors were displayed in 1*g* as well as in real and simulated µ*g*, but with different frequencies. Comparing the overall activity patterns shown during the two flight phases reveals that *H. incongruens* showed significantly (*Z* = 2.11, *p* = 0.035) more active behavior (crawling, swimming, and looping) in µ*g*, *N. monacha* was significantly (*Z* = 3.41, *p* = 0.001) more passive (resting and drifting).

### Influence of microgravity and light on the orientation of different species

**Fig. 3:**
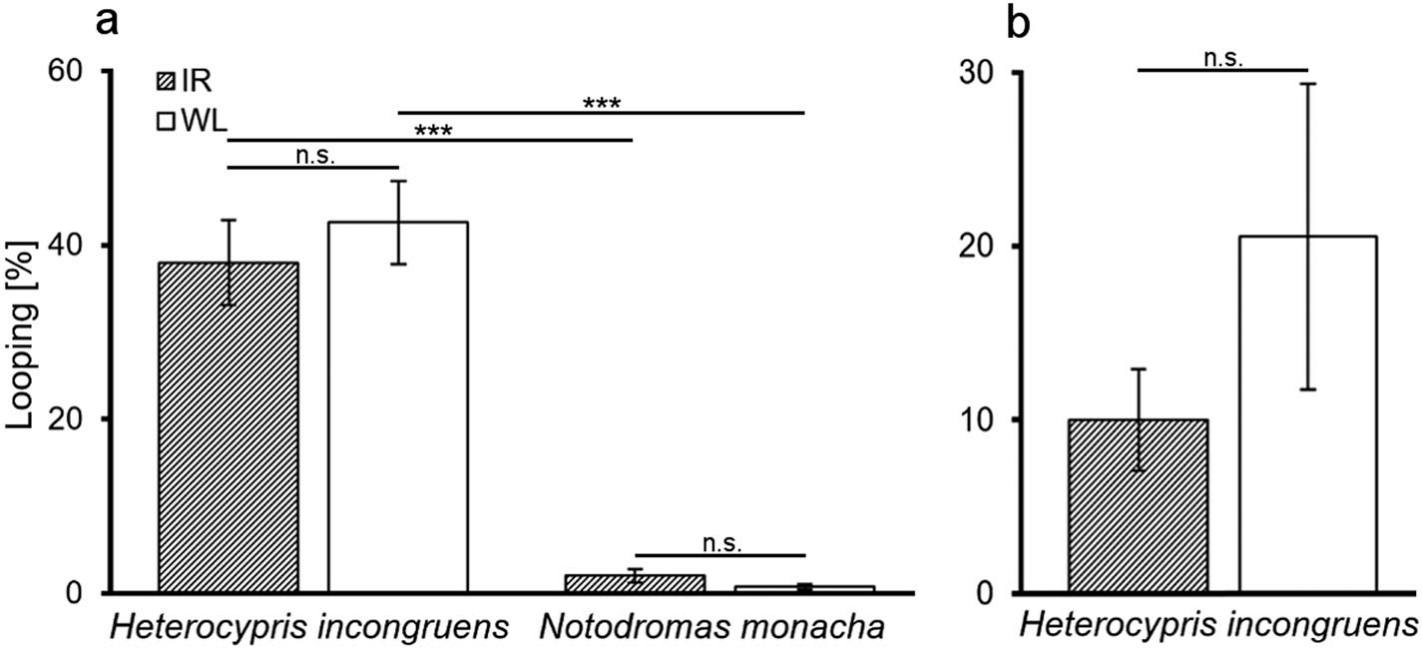
Relative looping durations (in %) in functional darkness (IR illumination) and under WL illumination during **a** parabolic flights (*H. incongruens* and *N. monacha*, *n* = 18, except for *H. incongruens* in WL: *n* = 12) and during **b** clinorotation (*H. incongruens*, *n*_IR_ = 10, *n*_WL_ = 7). Mean value ± SEM, levels of significance: ***p ≤ 0.001, n.s. = not significant

*H. incongruens* looped in IR as well as in WL significantly longer than *N. monacha* during the PFs (*U*_IR_ = 7.0, *p* < 0.001; *U*_WL_ = 0.0, *p* = < 0.001). Within each species, the looping durations did not significantly differ between the illumination modes (*H. incongruens: Z* = 0.63, *p* = 0.530; *N. monacha:* *Z* = 1.24, *p* = 0.214; Fig. 3a).

Since the ostracods were exposed to the identical clino typic interferences (see section above and Fig. 1b), both, during the trials in IR and in WL (in contrast to the comparison between 1*g* und rotation), a reasonable comparison of looping behavior between the illumination modes was possible. Just like during the PFs, the relative looping durations did not significantly differ between IR and WL (*U* = 43.0, *p* = 0.48; Fig. 3b).

### Acclimation to microgravity

For both species, all slopes of the trendlines, which were fitted through the looping durations of the consecutive µ*g* intervals are almost horizontal with the numeric values close to zero.

**Fig. 4:**
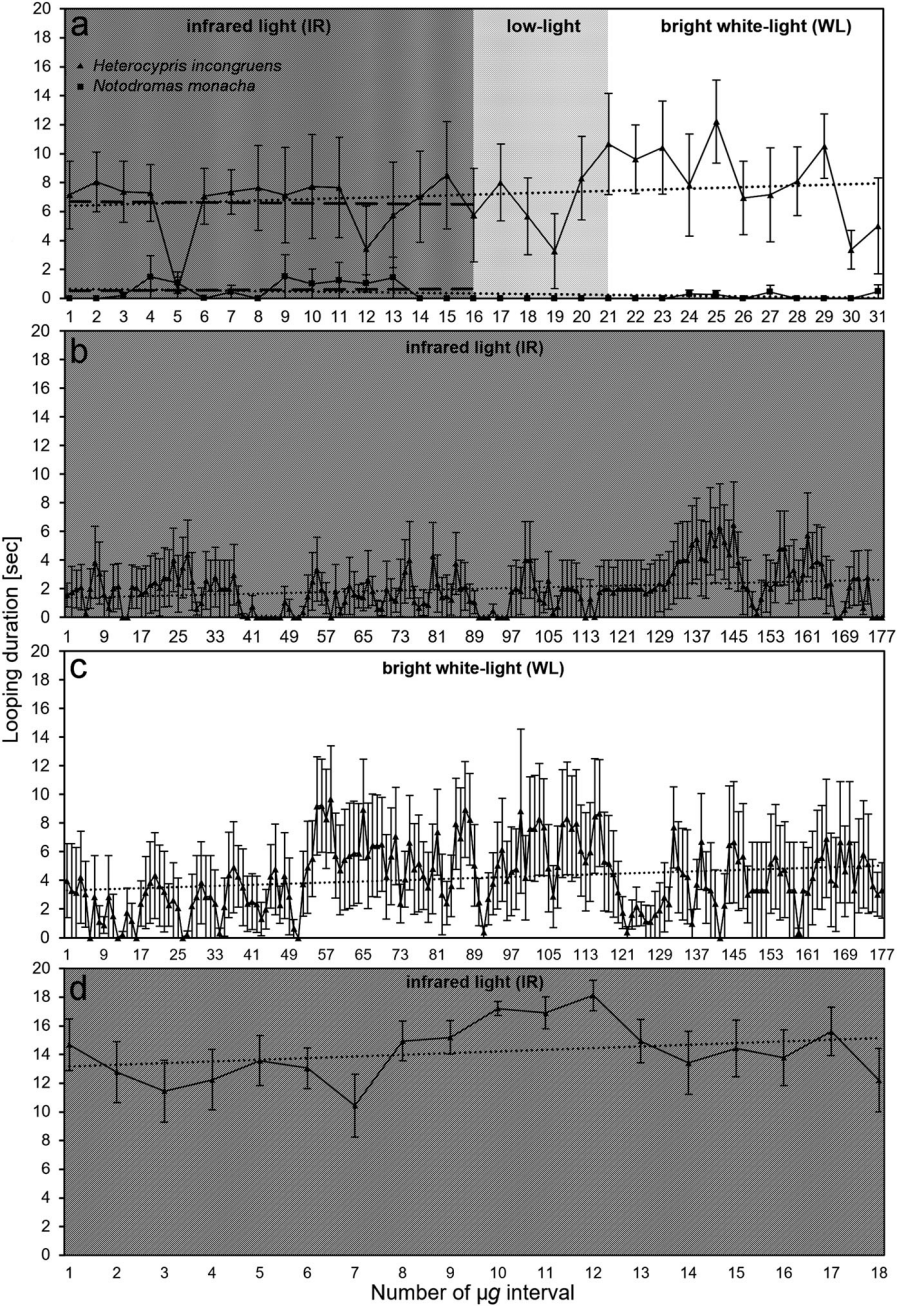
Duration of looping behavior (in s) of **a**, **b**, **c**, **d** *H. incongruens* and **a** *N. monacha* shown during 20 s long µ*g* periods achieved during **a** parabolic flights (*n* = 12, except for Ps 8–31 in *H. incongruens*: *n* = 6), **b**, **c** clinorotation (*n*_b_ = 10, *n*_c_ = 7), and **d** a TEXUS sounding rocket flight (*n* = 10). Dotted trendlines (…) are fitted through the data points of all µ*g* periods achieved per method, dashed trendlines (– – –) through the data points of the parabolic flight µ*g* intervals in functional darkness (IR illumination). Mean value ± SEM

However, the trendlines fitted through the data points measured under IR illumination during the PFs and those of the whole flights are contradicting (Fig. 4a). In *H. incongruens* the slope of the trendline through the IR data is negative (*β* = −0.014), whereas that of the whole PFs is positive (*β* = 0.051). In *N. monacha*, the opposite is the case (*β*_IR_ = 0.009; *β*_wholePF_ = −0.021).

Both trendlines fitted through the looping durations of the consecutive simulated µ*g* intervals (clino) under IR (*β* = 0.007) as well as WL (*β* = 0.010) illumination are almost horizontal, but slightly ascending (Fig. 4b, c). The same is true for the TEXUS trendline, which is, however, ascending a little steeper than all others (*β* = 0.116, Fig. 4d).

### Threshold of gravity perception

**Fig. 5:**
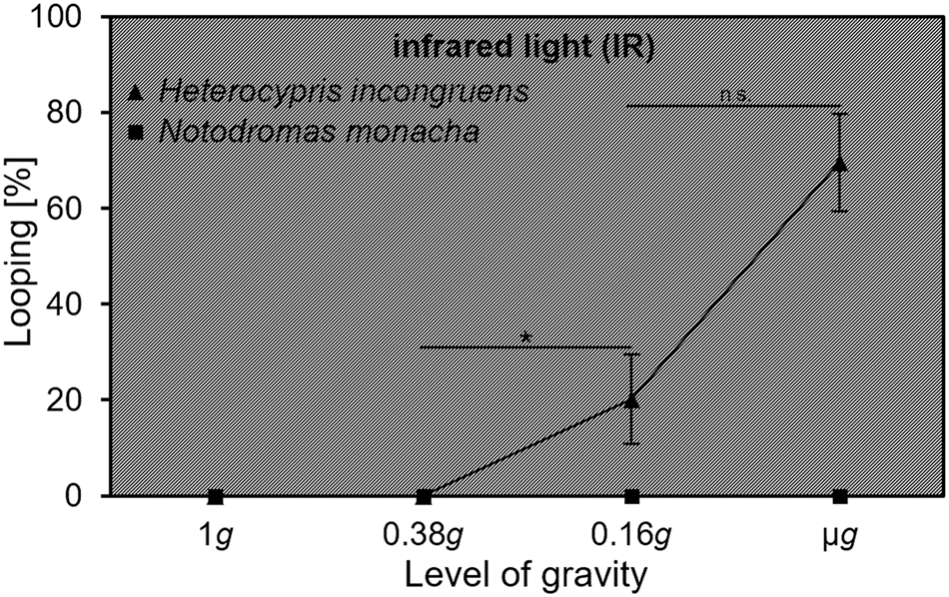
Relative looping durations (in %) of *H. incongruens* and *N. monacha* during different levels of gravity, achieved during parabolic flights (under IR illumination). Mean value ± SEM, *n* = 6, levels of significance: *p ≤ 0.05, n.s. = not significant (comparisons between data points with the same value are neglected)

Both species neither looped in 1*g* nor at 0.38*g*. At a gravity level of 0.16*g*, the relative looping duration of *H. incongruens* increased significantly in comparison to 1*g* and 0.38*g* (*χ*^2^(1, *n* = 6) = 4.0, *p* = 0.046) and already four out of the six individuals looped. The maximum relative looping duration as well as the number of looping *H. incongruens* occurred in µ*g*, as all six individuals looped. However, in comparison to 0.16*g*, this increase was not significant (*χ*^2^(1, *n* = 6) = 0.67, *p* = 0.414). *N. monacha* showed no looping at all during the consulted phases (Fig. 5).

## Discussion

### Microgravity-specific locomotion behavior and orientation

Our study reveals that in µ*g* the two ostracod species *H. incongruens* and *N. monacha* display looping behavior, which we never observed under 1*g* conditions (Fig. 1a). Since this behavior is a disorientation response,^27^ it can be concluded that both species perceive gravity and use it as cue for spatial orientation. The looping behavior displayed by *H. incongruens* under simulated µ*g* furthermore signifies that exposure to clino causes the same pattern of disorientation as real µ*g*. Hence, this method seems suitable to simulate the absence of the gravitational force for this species, and potentially also for other Ostracoda and organisms with similar gravity perception organs.

Drifting is the second behavior both species exclusively showed in µ*g*, but predominantly under simulated µg. Under real µg, the ostracods floated undirected and motionless through the water column with their fanned out first antennae protruding through a small gap of the ajar carapace. Under simulated µg, the behavioral pattern was the same, but the undirected nature of the drifting behavior was artificially altered, as clino induced non-gravitational effects next to the time-averaged nullification of gravity. The ostracods were forced on circular tracks due to the higher density of their body in contrast to the surrounding medium. In addition, they were passively aligned, since drag acts as tethering force stronger on the feathered first antennae, than on the smooth, compact carapace. As a result, the antennae constantly pointed to the center of the circle around which the animals rotated (Fig. 1b).

Although not observed in our experiments (probably due to the dimensions of the cuvettes and the resulting very short descending paths), the posture the ostracods take on when drifting is the same they also show in 1*g* at times. But since gravity acts on the animals under this condition, they descent with their posterior directed downwards and their first antennae upturned acting like a parachute.^33^ Descending then either stops, when the animals start to swim or reach the bottom, where they often stay for minutes (min) without moving (equivalent to resting behavior). Descending^33^ and also such prolonged resting phases (characterized as “playing dead”^29,34^) are assumed to be triggered by disturbance, stress, and adjustment to new conditions.^29,34^ Due to these reasons, we do not consider drifting as disorientation response in the absence of gravity although only shown in µ*g*. Hence, we exclusively deployed looping behavior for the analyses regarding orientation toward the gravitational vector.

### Orientational differences between the species

Compared to each other, *N. monacha* looped significantly less than *H. incongruens* in µ*g* (Fig. 3a). Two explanatory approaches may interpret this observation. The first says that the gravity perceiving mechanism of *N. monacha* is less sensitive than the one of *H. incongruens*. Orientation toward the gravitational vector could, as a consequence, be less important to *N. monacha* than to *H. incongruens*, explaining why the latter is more affected by its loss. Similar has been observed in fish (*Oryzias latipes*) with congenitally deficient vestibular organs. These fish are used to substitute orientation to gravity by relying on other senses (e.g., visual orientation), making them less dependent on gravity. Therefore, they spent far less time looping than conspecifics with intact vestibular organs when exposed to µ*g* (during PFs.^35^)

The second explanation again includes the unequal sensitivity of gravity perceiving mechanism of different ostracod species. But in contrast to the first one it says that *N. monacha* features the gravity perceiving mechanism with the higher sensitivity. This assumption is supported by the differing thresholds of gravity perception: ~66% of the *H. incongruens* individuals looped in 0.16*g*, but none in 0.38*g* (Fig. 5), revealing a perception threshold in-between these two values. *N. monacha*, however, did not loop at all during the threshold experiment and only approximately 2% of the µ*g* time during the other experiments in functional darkness (IR illumination). Hence, this species gravity perceiving mechanism might be sensitive enough to still perceive the ~10^−2^*g* achieved during PFs most of the time. A reason for a possible higher sensitivity of the gravity perceiving organ of *N. monacha* in contrast to *H. incongruens* could be their differing annidation within their habitats. Both species are good swimmers and move with their ventral side directed downward through the water column. *N. monacha* spends furthermore considerable amounts of time upside down with the ventral side attached to the water surface membrane.^30^ Maintaining and switching between these diametrically opposed body positions are more complex behavioral patterns than mere swimming behavior and demand a higher level of postural control,^21^ and possibly a more sophisticated or delicate gravity perception.

However, except from the fact that ostracods do not feature classical statocysts,^36^ nothing is known about their gravity perceiving organs or mechanisms. Therefore, the reasons for the unequal looping durations and the differing thresholds of gravity perception of the two species remain speculative.

### Possible reduction of disorientation in microgravity—acclimation over time and light as orientational cue

The use of Ostracoda in BLSS for space exploration might be interfered by µ*g*-caused behavioral changes that could prejudice their normal life cycle. Whereas this issue will probably not become important in *N. monacha*, as this species barely looped (Fig. 3a) and showed a generally decreased activity in µ*g* (Fig. 2), the opposite might be the case in *H. incongruens*. Looping behavior made up one of the largest proportions of this species’ total behavior (~40% (Fig. 3a) to ~70% (Fig. 4d)) and was thus linked to a distinctly increased overall activity (Fig. 2). A reduction of these changes would thus be desirable, as it is so far not known, if for example the animals’ metabolic rate,^37^ energy consumption, or aging^38^ are influenced by the altered activity patterns.

Regarding acclimation to µ*g* over time, i.e., a decrease of looping behavior, our study reveals a lack in both species. In *H. incongruens* neither during exposure to several min of uninterrupted real µ*g*, nor during approximately 1 h of simulated µ*g* (Fig. 4b, c, d), a reduction could be observed. During the PFs indeed, a slight decrease of the looping behavior was found, when only considering the flight time in functional darkness, but when considering the whole flight, looping slightly increased (Fig. 4a). In general, the slopes of all trendlines, regardless of which method was used, are close to zero, indicating constant looping of *H. incongruens* over time. Also *N. monacha* probably does not acclimate to µ*g*, as the slopes of its’ looping trendlines are also close to zero (Fig. 4a). In this case, however, acclimation was only tested during PFs. However, all methods utilized to investigate acclimation of *H. incongruens* yield the same outcome and acclimation over PFs has been observed in other organisms, as well.^39,40^ It is thus probable that the results gained using PFs are reliable. These findings are in line with the observation that the unidentified ostracod species on MIR space station did not acclimate during a 4-months sojourn. Until the end of this long-term experiment, consistently around 50% of the animals showed looping behavior.^22,23^ Considering all results, we assume that Ostracoda do not habituate to µ*g* in terms of a reduction of the shown kinetotic behavior.

Apart from acclimation over time, disorientation may also be reduced by substituting orientation along the gravitational vector with another orientational cue. Many animals, such as fish^41^ and also other crustaceans (e.g., *Daphnia,*^42^) use the direction of light in addition to gravity for orientation and postural control. Since light has also been found to influence the behavior of Ostracoda,^31,32^ directed WL could provide an orientational cue in µ*g*.

However, our experiments reveal that photo-orientation barely compensates µ*g*-caused disorientation. Neither *H. incongruens* nor *N. monacha* showed differing looping durations between functional darkness and WL illumination (Fig. 3a, b). And also during the aforementioned MIR study, where light was permanently powered on during sojourn in space, looping was the most observed behavior.^22,23^ These results are not only important regarding orientation in µ*g*, but also with respect to the necessity of the operation and period of illumination within a BLSS, as artificial illumination is an energy-consuming factor, which might be avoided.

## Conclusion

At first sight, the absent reduction of looping behavior and thus disorientation in µ*g*, whether through acclimation or utilization of light as orientational cue, renders Ostracoda, unqualified for BLSS. However, even the highly looping species *H. incongruens* shows a similar life-history parameters and foraging and feeding behavior as under 1*g* conditions^18,43^ and also the ostracods on MIR space station survived and reproduced successfully for several months in space, despite of the continuous looping behavior.^22,23^ Therefore, we assume that Ostracoda are still promising candidates for BLSS with an only slightly or unimpaired performance in µ*g* on the long run. Thereby, it should be assessed in future experiments, if barely looping ostracod species, such as *N. monacha*, are better qualified for BLSS on the long run, since they may have a lower energy consumption and show a more natural locomotion and orientation in space.

## Material and methods

### Animal culture

*H. incongruens* and *N. monacha* were captured from a small ephemeral pond near Munich, Germany (GPS-coordinates: 48°06′33.9″N 11°27′25.6″E) and cultured in a climate chamber (20 ± 0.5 °C under neon light at a constant photoperiod, 15 h day: 9 h night). The ostracods were kept in semi-artificial medium (for composition see ref. ^44^) with detritus from the pond of origin added as substrate and fed weekly with TetraPhyll fish food (Tetra GmbH, Germany). During the experiment campaigns, they were kept without substrate in Volvic mineral water (Danone Waters Deutschland GmbH, Germany) at room temperature (20 ± 2 °C) and fed as usual.

The experiments were performed in accordance with the German Animal Welfare Act (TierSchG) and the EU-Guideline 86/609/EEC for the protection of animals in experimentation and approved by the Ethics Committee at the University of Bayreuth.

### Low-gravity platforms

#### Parabolic flights

Periods of low gravity within an aircraft can be achieved by aviating parabolic trajectories. In the first phase of such a maneuver, the aircraft ascents steeply from horizontal flight at an angle of 47° whereby hypergravity (~1.8*g*) occurs. Engine thrust is reduced to the extend required to compensate for the air drag and the aircraft proceeds in free fall mode. During this period of the parabola (P), µ*g* (~10^−2^*g* for 20–23 s) is achieved. For partial-gravity conditions, the Ps are aviated flatter injection angles (42° to achieve 0.16*g* for ~25 s during a so-called Moon-P, or 38° to achieve 0.38*g* for ~32 s during a Mars-P). In order to escape the free fall or partial-gravity mode, the engine thrust is increased and the nose of the aircraft is pulled up again, which causes another hypergravity period before returning to horizontal flight (for details, see refs. ^45^^,^^46^).

The experiments were carried on 6 flight days (FDs): four regular FDs (RFD I-IV) with 31 µ*g*-Ps each, and two additional FDs (AFD I and II) with one Moon-P and one Mars-P prior to 13 µ*g*-Ps each. *H. incongruens* was deployed on RFD I and III and on AFD I, *N. monacha* on RFD II and IV and on ADF II. On RFD III a hardware error occurred, resulting in an experiment abort after P 7 (Table 1). New naive, randomly selected individuals were used on each FD in order to exclude any habituation or conditioning effects. However, the animals could not be replaced during flight, thus same individuals were recorded during all flight phases and experimental conditions on a single FD.

**Table 1:** Number of periods and respective total durations of the µ*g* and 1*g* periods (partial gravity not included) for each ostracod species, which were considered for the analyses

		*H. incongruens*		*N. monacha*	
		IR	WL	IR	WL
Parabolic flights	RFD I	µ*g*: 16 = 320 s	µ*g*: 10 = 200 s	–	–
		1*g*: 16 = 320 s	1*g*: 10 = 200 s		
	RFD II	–	–	µ*g*: 16 = 320 s	µ*g*: 10 = 200 s
				1*g*: 16 = 320 s	1*g*: 10 = 200 s
	RFD III	µ*g*: 7 = 140 s	Hardware defect	–	–
		1*g*: 7 = 140 s			
	RFD IV	–	–	µ*g*: 16 = 320 s	µ*g*: 10 = 200 s
				1*g*: 16 = 320 s	1*g*: 10 = 200 s
	AFD I	µ*g*: 5 = 100 s	µ*g*: 8 = 160 s	–	–
		1*g*: 5 = 100 s	1*g*: 8 = 160 s		
	AFD II	–	–	µ*g*: 5 = 100 s	µ*g*: 8 = 160 s
				1*g*: 5 = 100 s	1*g*: 8 = 160 s
Clinorotation	Trials 1–10	µ*g*: 177 = 3540 s	–	–	–
		1*g*: 60 = 1200 s			
	Trials 11–17	–	µ*g*: 177 = 3540 s	–	–
			1*g*: 60 = 1200 s		
TEXUS		µ*g*: 18 = 360 s	–	–	–
		1*g*: –			

Furthermore, the utilized low-gravity platform (parabolic flights with number and mode of flight days [RFD I-IV and AFD I-II] and clinorotation with number of considered trials) and the illumination mode (IR or WL) are indicated

Furthermore, different illumination modes were applied in the course of the flights. During the first 16 Ps on the RFDs and 7 Ps (including partial-gravity Ps) on the AFDs, IR spots (wavelength peak = 940 nm, TV6816, ABUS, Germany) were used, emitting light outside the visible spectrum (functional darkness) but within the sensitivity of the camera (1/3″ A1 Pro b/w board camera, Conrad Electronic SE, Germany), which recorded the ostracods. After the IR mode, five Ps under low-light (single WL LED) followed on the RFDs (for a smooth transition between IR and bright WL, not utilized for any analysis). On the AFDs, no low-light was applied due to the low number of Ps on these FDs. During the last 10 Ps on the RFDs and the last 8 Ps on the AFDs, bright WL spots (2700 Kelvin, L1325 Dioder, IKEA GmbH & Co. KG, Germany), mounted above the cuvettes, were powered on.

Before each flight, two cuvettes (30 mm × 30 mm × 10 mm, reamed out of Makrolon, sealed with a Plexiglas cover plate) were filled air bubble-free with Volvic mineral water (to avoid interference of the µ*g *quality by drifting bubbles) and three adult ostracods each were inserted. In order to assure a sufficient oxygen supply during the approximately 3 h of total flight time per FD, the cuvettes were piped allowing a regular medium replacement using a fuel gear pump (Modelcraft, Conrad Electronic SE, Germany).

The experimental hardware was built and integrated in the PF aircraft (Airbus A300 Zero-G) in accordance with the “Experiment Safety Data Package” of the operating company (Novespace, France) and approved by the Direction générale de l’armement Essais en Vol (DGA EV, France).

For the analyses, 20 s around the median of each µ*g* period (or 25 or 32 s of the respective partial-gravity periods) per P (treatment) and of the subsequent horizontal flight phase (1*g* control) were used. During approximately 3% of this time single ostracods could, due to reflections within the cuvettes or low contrast of the recordings, not be detected on the videos. As these events were evenly distributed among the animals, they were not considered in the analyses.

#### TEXUS sounding rocket

During the flight of a TEXUS sounding rocket, a continuous, approximately 360 s long µ*g* (10^–4^*g*) period occurred. Similar to the PFs, µ*g* is achieved during the free fall phase of the parabolic trajectory, the rocket passes through (for details on conditions and flight maneuver, see ref. ^47^). However, a 1*g* control could not be recorded. Horizontal flight does not take place before or after the P and a control on ground was not deemed reasonable, as vibrations and other disturbance only occur in flight. Furthermore, no WL was applied in order to achieve the longest possible µ*g* period under constant illumination.

The experimental hardware included four cylindrical cuvettes (depth: 55 mm, diameter: 35 mm, reamed out of Makrolon and sealed with a Plexiglas cover plate), into which three randomly selected adult *H. incongruens* each were inserted. They were illuminated using IR arrays (wavelength peak = 940 nm, IR67-21C/TR8, Everlight Electronics Co Ltd., Taiwan) mounted underneath the cuvettes and filmed using a camera (XC-ST70CE, Sony Europe Limited, Great Britain, equipped with IR permeable objective Apo-Xenoplan 1.8/35, Jos. Schneider Optische Werke GmbH, Germany). As space was very limited within the rocket, the four cuvettes were recorded by one only camera. Thus, a blind angle and hence a masked area arose within each cuvette. Two of the in total 12 ostracods spent more than 3% of the µ*g* period within such an area, thus they were excluded from the analysis.

#### Clinorotation

Clinostats rotate samples horizontally (uniaxial) at constant speed to guarantee a continuous reorientation. In doing so, gravity is neither eliminated nor altered, but it is assumed that biological systems do not perceive the gravitational stimulus, when reoriented fast enough.^24,48^

In order to expose *H. incongruens* to simulated µ*g*, single, randomly selected, adult animals were inserted into a cylindrical cuvette (depth: 5 mm, diameter: 22 mm, reamed out of Plexiglas, sealed with a glass cover plate) which was rotated with 60 revolutions per minute using a random positioning machine (Dutch Space, The Netherlands) in 2D mode (rotation of only one arm, residual gravity of ~0.04*g* at the periphery of the cuvette, calculation, see ref. ^49^) For the 10 experimental trials under IR (wavelength peak = 880 nm, 26 mm LED Lamp Cluster, Kingbright, Germany, mounted behind the cuvette) and the 7 under WL illumination (14,000 mCD white LED, Nichia, Japan, mounted on top of the cuvette and jointly rotated with it to achieve a directed illumination despite the rotation) always new, naive ostracods were used. They were recorded using a board camera (CMOS-mini-camera-module, Conrad Electronics, Germany).

Each experimental trial included a 10-min long settling-in phase, an ensuing 20 min long 1*g* control phase and an 1-h long clino phase. For the analysis, the first min after start of the rotation was neglected as it took this long to reach the best possible alignment of the angular speed of the medium adjacent to the cuvette surface through to the rotation center.

### Encoding of locomotion behavior and statistical analysis

In order to enable the analysis of locomotion and orientation behavioral categories, comprising every possible locomotion behavior of the two species, were defined:

- Crawling—animals are active on the inner surfaces of the cuvette
- Resting—animals are inactive on the inner surfaces of the cuvette
- Swimming—animals are active in the water column (but not in circular motion), and do not touch the cuvette surfaces
- Drifting—animals float inactive through water column, and do not touch the cuvette surfaces
- Looping—animals somersault/swim actively in a circular motion

Using the event logging software Observer XT 10 (Noldus, The Netherlands), the video recorded ostracods were individually tracked and displayed locomotion behavior was end-to-end classified into the defined categories (referred as to behaviors in the following). During this classification, the experimenter was blinded to the gravity conditions, as they could not be recognized in the video recordings.

For the analyses (except for acclimation to µ*g*), non-transformed relative durations^50^ of the displayed behaviors were used, as the phases during which the different gravity levels occurred differed in duration between the used methods. Furthermore, not always the same number of Ps occurred on the six FDs of the PFs (due to hardware error or type of FD: RFD or AFD).

The subsequent statistical analysis was conducted using SPSS Statistics (version 22.0 for Windows, IBM Corporation, USA), except for linear regression (trendline analysis), which was conducted using Excel (version 2013 for Windows, Microsoft Corporation, USA). Normal distribution was tested by Shapiro–Wilk tests, homoscedasticity by Levene tests. As these assumptions for parametric testing are not met, non-parametric tests were utilized. The levels of statistical significance are designated in the respective graphs.

### Comparison of locomotion behavior between normal and microgravity

The alterations of locomotion and orientation between µ*g* and 1*g* could be revealed by comparing the relative durations of looping and drifting behavior recorded during the respective flight phases of the PFs (Wilcoxon-signed-rank tests). Furthermore, the overall activity during these flight phases was calculated from the summed relative durations of all active (crawling, swimming, and looping) and passive (resting and drifting) behaviors and compared using Wilcoxon-signed-rank tests. In order to investigate pure orientation by gravity without any influence of light, only behavior performed under IR illumination was utilized (total duration of a behavior in µ*g* or 1*g* under IR/total duration of respective flight phase under IR, Table 1).

### Influence of microgravity and light on the orientation of different species

In order to reveal differences between the orientation capacities of *H. incongruens* and *N. monacha*, the looping behavior shown in µ*g* during the PFs (WL and IR illumination) was utilized (relative durations compared using Mann–Whitney U tests). The relative looping durations observed under the two illumination modes, during the PFs and also during clino, were furthermore used to disentangle the interaction of gravity and light in orientation (PFs: Wilcoxon-signed-rank tests, as the same individuals were recorded on the single FDs; clino: Mann–Whitney U tests, as different animals were used in each experimental trial, Table 1).

### Acclimation to microgravity

Despite of the 1*g* and hypergravity periods between the µ*g* periods, acclimation to µ*g* has already been observed over PFs.^39,40^ Thus, we further utilized these flights (WL and IR illumination) to investigate possible acclimation of *N. monacha* and *H. incongruens*. In order to verify these results and especially to extend the data on *H. incongruens* (few data usable due to a hardware error on RFD III), these species were further exposed to longer and uninterrupted µ*g* periods. During a TEXUS flight, the animals experienced approximately 360 s of µ*g* and during clino 59 min of simulated µ*g*, after assessing the suitability of the latter method by comparing the shown behaviors to those observed under real µ*g*. In order to make these uninterrupted µ*g* periods comparable to the PF data (consisting of always a ~20 s long µ*g* interval per P), we split the clino and TEXUS µ*g* periods into the respective number of 20 s long intervals (Table 1). After that, trendlines (least squares lines) were fitted through the duration of looping behavior of each of the resulting consecutive µ*g* intervals and their slopes were calculated.

### Threshold of gravity perception

The thresholds of gravity perception (onset of disorientation) of both species was approached by analyzing the relative looping durations shown during the reduction of the gravitational force from 1*g* over 0.38*g* and 0.16*g* to µ*g* during the AFDs of the PFs (Friedman tests).

### Data availability

The data that support the findings of this study are available from the corresponding author upon request.
